# Venous Thromboembolism Prophylaxis in Medical and Surgical Patients – What’s Our Reality?

**DOI:** 10.7759/cureus.49444

**Published:** 2023-11-26

**Authors:** Diana Rocha, Carolina Tintim Lobato, Diogo Melo Pinto, Francisca Marques, Tatiana Marques, Carolina Guedes

**Affiliations:** 1 Internal Medicine, Hospital Pedro Hispano, Matosinhos, PRT; 2 Intensive Care Unit, Hospital Pedro Hispano, Matosinhos, PRT; 3 General Surgery, Hospital Pedro Hispano, Matosinhos, PRT; 4 Endocrinology, Hospital Pedro Hispano, Matosinhos, PRT

**Keywords:** risk assessment, enoxaparin, pulmonary embolism, venous thrombosis, venous thromboembolism

## Abstract

Introduction: Venous thromboembolism (VTE) includes pulmonary embolism (PE), deep vein thrombosis (DVT) in lower limbs, and thrombosis in rare locations. VTE is a common cardiovascular disease, being the leading preventable cause of in-hospital death. Both surgical and acute medical patients have an elevated risk of developing VTE. VTE risk assessment is essential to identify patients who might benefit from VTE prophylaxis accurately. Clinical data on risk factors and prophylaxis in Portugal are scarce. We aimed to determine the proportion of at-risk patients who received prophylaxis and the incidence of bleeding events. We also intended to study the rate of VTE in a cohort of medical and surgical patients during the hospitalization period and three months after discharge.

Methods: During one week in 2020, adults admitted for more than 72hr to a medical or surgical ward were included. The study excluded patients with a diagnosis of VTE three months before hospitalization and who were either chronically receiving anticoagulation therapy or had started it 48 hours after admission. Risk assessments were based on the Padua Prediction Score (PPS) for medical patients and the Caprini Risk Assessment Model (CRAM) for surgical patients. We used CHEST guidelines, 9th edition, to determine the adequacy of the prophylactic method.

Results: A total of 123 patients were analyzed, 18.7% of which tested positive for SARS-CoV-2. VTE risk in surgical patients was categorized as very low or low (16.6%), moderate (37.5%), and high (43.8%), according to the CRAM. Risk in medical patients was categorized as low (60.0%) or high (40.0%) according to the PPS. We estimated that VTE chemoprophylaxis was overused in about 30.0% of patients vs. 7.0% who were at risk and did not receive appropriate chemoprophylaxis. The rate of thromboembolic events was 4.1% (n=5), 2 of which happened after discharge. Two of these patients were under VTE prophylaxis during hospitalization. Major bleeding occurred in 2.4% of patients (n=3).

Discussion: A significant number of hospitalized patients are deemed to be at risk for VTE, making appropriate prophylaxis essential. The results emphasize the insufficient management of VTE prophylaxis.

## Introduction

Venous thromboembolism (VTE) comprises deep vein thrombosis (DVT), pulmonary embolism (PE), and thrombosis in atypical localizations. Risk factors for VTE are divided into strong, moderate, and weak factors [1-3]. Strong risk factors include major surgery, prolonged immobilization, major trauma, and active cancer, but in most events, only weak risk factors are identified or none [3]. Venous thromboembolism affects over 1 in 1000 adults worldwide annually [4] and contributes greatly to cardiovascular morbimortality and healthcare costs [3,5-7]. It affects about 5.0% to 15.0% of patients hospitalized for medical or surgical reasons [8]. Hospital-acquired VTE is a consequence of hospitalization, disease, and/or treatment and can occur during hospital stay and up to 90 days post-discharge [1].

Adequate and timely use of thromboprophylaxis in surgical and medical patients at risk is recommended by international guidelines to decrease the incidence and global burden of Hospital-acquired VTE [1,3,6,9-12]. The effectiveness and safety of pharmacological interventions were proven in clinical trials. When anticoagulation is contraindicated, mechanical interventions might be used [3,6]. It is also recommended to implement validated risk assessment models to aid the stratification of hospitalized patients according to thrombotic and bleeding risk. Although with inherent limitations and varied performances, these clinical tools help identify those patients who would benefit from in-hospital thromboprophylaxis [1,6,9,10].

In 2008, a large international study reported that 51.8% of hospitalized patients (both surgical and medical) were at risk of VTE; however, only 58.5% of at-risk surgical patients and 39.5% of at-risk medical patients received American College of Chest Physicians (ACCP) recommended VTE prophylaxis [7]. In Portugal, the ENDORSE study revealed that 52.7% of all hospitalized patients were at risk of VTE. However, the rate of adequate thromboprophylaxis was only 58.5%, and more than a third of patients, although unsuitable, were offered thromboprophylaxis inappropriately [7,13]. A more recent study also revealed that even though a substantial proportion of medical and surgical patients were at risk for VTE, implementation of prophylaxis was low, particularly in medical patients [8]. In fact, despite the proven clinical benefit and existing recommendations, thromboprophylaxis is still underused, especially in medical patients around the globe and with clear geographical differences [1,3,5-8,10,14]. Clinical data on VTE risk and thromboprophylaxis management in Portugal are scarce.

In this study, we aimed to estimate the risk of VTE in a group of medical and surgical inpatients and evaluate the adequacy of thromboprophylaxis and the incidence of bleeding events. We also intended to determine the rate of VTE during hospitalization and 90 days post-discharge.

## Materials and methods

This prospective observational descriptive study was conducted at Pedro Hispano Hospital, a secondary care unit in northern Portugal. We retrieved and evaluated all medical records regarding all adults (> 18 years) admitted for more than 72 hours to a medical and surgical ward during the first week of May 2020.

All patients with a diagnosis of VTE within the last 3 months or who were either chronically receiving anticoagulation therapy or had started it in the first 48 hours after admission were excluded. Patients admitted to urology, otorhinolaryngology, and ophthalmology wards were also excluded.

The data was extracted using a standardized form, collecting all information needed regarding socio-demographic characteristics, previous medical history, current admission, prescribed prophylaxis of VTE or reported reasons for thromboprophylaxis omission, incidence of VTE within the first 90 days after discharge, and bleeding events. Venous thromboembolism risk assessment for medical patients was based on the Padua Prediction Score [15] and the Caprini Risk Assessment Score [16] for surgical patients. We used the American College of Chest Physicians (ACCP) evidence-based consensus guidelines to determine the adequacy of the prophylactic method [17,18].

We performed subgroup analyses regarding patients admitted to a surgical or medical ward. A VTE comprises both episodes of PE or DVT that were clinically suspected, registered in clinical files, and confirmed via Doppler ultrasound or Computed Tomography (CT) angiography. We investigated the frequency of patients classified at low, moderate, or high risk of VTE, the rate of thromboprophylaxis used in each group, and suitable thromboprophylaxis among each subgroup. We also determined the rate of VTE during hospitalization and up to 90 days post-discharge. Furthermore, we collected data regarding bleeding outcomes, defined according to the International Society on Thrombosis and Haemostasis (ISTH) definitions, of both major bleedings (fatal and/or symptomatic bleeding in a critical area or organ, such as intracranial, intraspinal, intraocular, retroperitoneal, intra-articular or pericardial, or intramuscular with compartment syndrome; bleeding causing a fall in hemoglobin level greater than 2g/dL or leading to a transfusion of 2 or more units of whole blood or red cells). Conversely, clinically relevant non-major bleeding (CRNMB) is considered overt bleeding, not meeting the criteria of major bleeding but associated with medical intervention [19,20].

Statistical analysis was performed using Microsoft Excel and SPSS Statistics Version 26.0 from IBMÒ. A descriptive analysis of collected data was conducted. Categorical variables are presented as frequencies and percentages, while continuous variables are expressed as means and standard deviations due to their normal parametrical distribution. Normal distribution was checked using skewness and kurtosis. We transformed some continuous variables into dichotomic data according to international scores (such as BMI). All reported p-values are two-tailed, with a p-value < 0.05 indicating statistical significance.

The Hospital's Ethics Committee approved this study before data collection. Patients' written informed consent was collected for access to their medical records. Anonymity and confidentiality were assured.

## Results

Baseline characteristics

**Figure 1:**
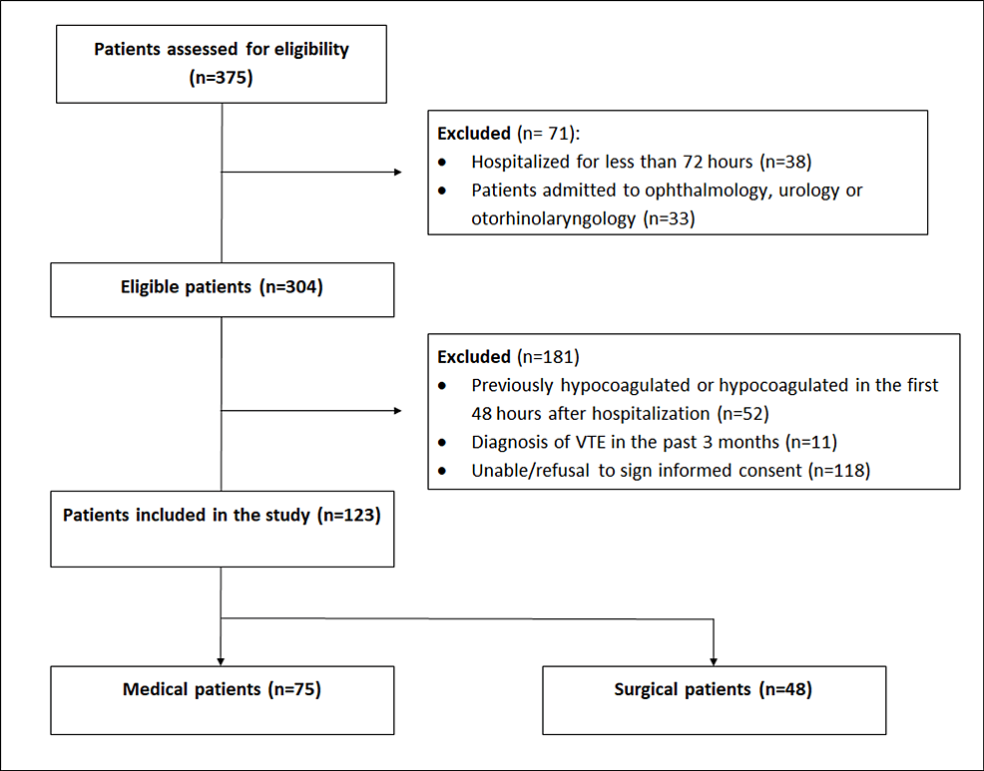
Study population selection.

From 375 eligible patients, 123 patients, 75 medical patients, and 45 surgical patients (figure 1) were included in the study sample.

The study sample's mean age was 68.2 ± 1.3 years; the majority were male (55.7%). Medical patients were mostly male (60.0%) and had a mean age of 70.6 ± 1.5 years. Approximately half of surgical patients were male (48.9%) and had a mean age of 64.4 ± 2.1 years. Most patients were admitted to the hospital in an urgent context (90.2% of the total sample, 98.7% of medical patients, 77.1% of surgical patients). Patients' socio-demographic characteristics, previous medical history, and hospitalization characteristics are presented in Table 1.

**Table 1:** Baseline Characteristics of Patients in Medical and Surgical wards SD - Standard Deviation. BMI - Body Mass Index. *1 missing value for male sex 1 This definition does not include thrombophlebitis or superficial venous thrombosis 2 Defined as bedrest or not walking during hospitalisation

Table 1. Characteristics of Patients						
Characteristics of Patients	All		Medical patients		Surgical patients	
	(n=123)		(n=75)		(n=48)	
Age - mean ± SD – yr	68.2	± 1.3	70.6	± 1.5	64.4	± 2.1
Male sex - no. (%)*	68	(55.7)	45	(60.0)	23	(48.9)
Previous Medical History - no. (%)						
Previous venous thromboembolism^1^	5	(4.1)	4	(5.3)	1	(2.1)
Thrombophilic conditions	0	(0.0)	0	(0.0)	0	(0.0)
Heart failure and/or respiratory insufficiency	30	(24.4)	21	(28.0)	9	(18.8)
Chronic kidney disease	14	(11.4)	9	(12.0)	5	(10.4)
Rheumatologic disorder	8	(6.5)	2	(2.7)	6	(12.5)
Obesity (BMI ≥ 30kg/m^2^)	29	(23.6)	18	(24.0)	11	(22.9)
Ongoing hormonal treatment	0	(0.0)	0	(0.0)	0	(0.0)
Antiaggregant therapy	30	(24.4)	23	(30.7)	7	(14.6)
Chronic thrombocytopenia (Platelets < 50× 10^9^/L)	3	(2.4)	1	(1.3)	2	(4.2)
Active cancer	28	(22.8)	15	(20.0)	13	(27.1)
Surgery and/or trauma in the previous month	6	(4.9)	2	(2.7)	4	(8.3)
Type of admission - no. (%)						
Urgent	111	(90.2)	74	(98.7)	37	(77.1)
Elective	12	(9.8)	1	(1.3)	11	(22.9)
Characteristics of Hospitalization - no. (%)						
Acute myocardial infarct (current admission or in the previous month)	10	(8.1)	10	(13.3)	0	(0.0)
Stroke (current admission or in the previous month)	6	(4.9)	6	(8.0)	0	(0.0)
Acute kidney injury	26	(21.1)	18	(24.0)	8	(16.7)
Infection with or without sepsis	64	(52.0)	39	(52.0)	25	(52.1)
Reduced mobility^2^	14	(11.4)	6	(8.0)	8	(16.7)
SARS-CoV-2 infection	23	(18.7)	18	(24.0)	5	(10.4)

VTE risk stratification

**Figure 2:**
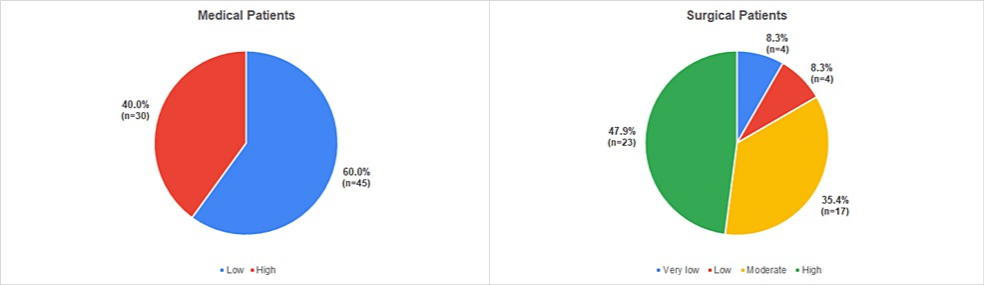
Risk assessment for VTE in Medical (n= 75) and Surgical (n=48) patients.

Risk for VTE in surgical inpatients was categorized as very low (8.3%), low (8.3%), moderate (35.4%) or high (47.9%) according to the Caprini Risk Assessment Score. Risk in medical patients was categorized as low (60.0%) or high (40.0%) according to the Padua Prediction Score (figure 2).

VTE prophylaxis

**Figure 3:**
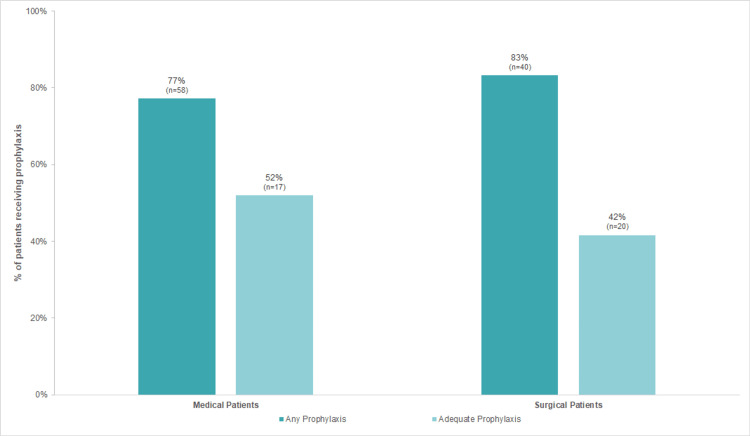
Adequacy of VTE prophylaxis in medical and surgical patients VTE: Venous thromboembolism

Venous thromboembolism prophylaxis was prescribed to 77.3% of medical and 83.3% of surgical patients (figure 3). The overall rate of use of the appropriate thromboprophylaxis method was registered in 48.0% of the sample, specifically in 52.0% of medical patients (26.7% of low-risk patients and 86.6% of high-risk patients) and in 41.7% of surgical patients (25.0% of very low-risk patients, 0.0% of low-risk patients, 82.4% of moderate-risk patients and 21.7% of high-risk patients). On the other hand, the overall rate of inappropriate choice of prophylaxis strategy was noted in 52.0% of patients (48.0% of medical patients and 58.3% of surgical patients).

Medical patients with a low VTE risk received no thromboprophylaxis in 26.7% of cases (the appropriate approach according to ACCP guidelines [17]). This group of patients received inappropriate mechanical and pharmacological thromboprophylaxis in 4.4% and 68.9% of cases, respectively. The overall rate of inappropriate prophylaxis in low-risk medical patients was 73.3%, with a significant overuse of pharmacological prophylaxis in this group.

Medical patients with a high risk of VTE received pharmacological thromboprophylaxis in 80.0% of cases, 3.3% received mechanical thromboprophylaxis due to elevated bleeding risk, and 3.3% received no prophylaxis due to contraindications for mechanical and pharmacological strategies. These three approaches are deemed appropriate according to ACCP guidelines [17], translating into an overall rate of adequate thromboprophylaxis in 86.6% of high-risk medical patients. On the other hand, 13.3% of high-risk TVE patients inappropriately didn't receive any type of prophylaxis without registered contraindications.

The appropriate prophylaxis strategy for surgical patients was defined according to ACCP guidelines [18]. Both surgical patients with a very low and low VTE risk received no thromboprophylaxis in 25.0% of cases and received pharmacological thromboprophylaxis in 75.0% of cases, neither appropriate strategies for this risk group. Surgical patients with a moderate VTE risk received pharmacological thromboprophylaxis in 82.4% of cases. In this group of patients, 17.6% of cases inappropriately didn't receive any type of thromboprophylaxis. Finally, surgical patients with high VTE risk were offered the appropriate thromboprophylaxis strategy in 21.7% of cases (17.4% received mechanical and pharmacological prophylaxis, and 4.3% received only mechanical prophylaxis due to high bleeding risk). The remaining patients received either only pharmacological prophylaxis (60.9%), only mechanical prophylaxis (4.3%), or none (13.0%).

The preferred pharmacological prophylaxis agent was low molecular weight heparin (LMWH) (100.0% of surgical patients and 87.3% of medical patients who received pharmacological prophylaxis). At the same time, fondaparinux was the prophylactic agent used in the remaining medical patients. Pharmacological prophylaxis was inappropriately used in 30.0% of patients (41.3% of medical patients and 12.5% of surgical patients). On the other hand, an underuse of mechanical thromboprophylaxis strategies was noted - used in only 4.0% and 4.2% of medical and surgical patients, respectively.

Outcomes

Table 2 shows bleeding and VTE events, mortality during hospitalization, and 90 days post-discharge. Venous thromboembolism occurred in 3 patients during the hospitalization period (2.4%), 2 surgical and 1 medical patient, none of which died during the study period. The surgical patients had either a moderate or high risk of VTE, and neither received the recommended thromboprophylaxis strategy. The medical patient had a low VTE risk and, accordingly, didn't receive mechanical or pharmacological prophylaxis. Two of three cases happened in patients who tested positive for SARS-CoV-2, one with mild symptoms and the other presented with pneumonia.

**Table 2:** Outcomes. VTE - Venous thromboembolism. *1 missing value for in-hospital VTE; 7 missing values for VTE < 90 days post-hospital discharge; **9 missing values for Death < 90 days post-hospital discharge. a Defined as a life-threatening hemorrhage or a symptomatic hemorrhage in a critical organ with an associated drop of >2g/dL in hemoglobin concentration or need for transfusion of ≥ 2 units of red blood cells. b Overt bleeding not meeting the criteria of major bleeding but associated with medical intervention c This definition does not include superficial venous thrombosis # Total of patients considered 117 (excluded patients that died during hospitalization)

Table 2. Outcomes						
In-hospital events						
	All		Medical patients		Surgical patients	
	(n=123)		(n=75)		(n=48)	
Bleeding events						
Major Bleeding^a^, n(%)	3	(2.4)	0	(0.0)	3	(6.3)
Clinically relevant non major bleeding^b^, n(%)	5	(4.1)	2	(2.7)	3	(6.3)
In-hospital VTE^c^, n(%)	3	(2.4)	1	(1.3)	2	(4.3)
In-hospital death, n(%)	6	(4.9)	5	(6.7)	1	(2.1)
Events < 90 days post-discharge						
	All^#^		Medical patients		Surgical patients	
	(n=117)		(n=70)		(n=47)	
VTE < 90 days post-hospital discharge*, n(%)	2	(1.8)	2	(3.0)	0	(0.0)
Death < 90 days post-hospital discharge**, n(%)	9	(8.3)	7	(10.4)	2	(4.9)

Two medical patients developed VTE on the 90 days following discharge; both had in-hospital low-risk VTE, and one of them inappropriately received pharmacological thromboprophylaxis.

We found no registry of major bleeding events in patients who received pharmacological thromboprophylaxis during hospitalization. Major bleeding was reported in only three surgical patients (6.3%) with high risk for VTE and high bleeding risk; these patients only used GCS and not pharmacologic strategies.

CRNMB was reported in three surgical patients (6.3%) and 2 medical patients (2.7%). The medical patients who developed bleeding events received pharmacological thromboprophylaxis despite having been considered low VTE risk. On the other hand, all 3 surgical patients shared an indication of pharmacological thromboprophylaxis (either for moderate or high risk for VTE) and no contraindication for receiving LMWH.

All-cause mortality during hospitalization and in the 90 days post-discharge was 4.9% and 8.3%, none of these deaths were related to VTE events.

## Discussion

Hospitalization for acute medical illness is associated with an eightfold increased risk of VTE [17], being the leading preventable cause of in-hospital death [21]. Conversely, the high incidence of postoperative VTE and the availability of effective methods of prevention mandate that thromboprophylaxis should be considered in every surgical patient [18]. Venous thromboembolism risk assessment is a cornerstone for accurately identifying patients who might benefit from VTE prophylaxis and consequent implementation of prevention strategies [22].

In our study, we found that the majority of admitted patients were at risk for VTE. Additionally, a large proportion of the patients received inappropriate prophylaxis strategy, which was both in the context of underuse or excessive prophylactic measures. The overall rate of the appropriate thromboprophylaxis method was 48.0%, slightly less than described in other studies [7,8,13].

Concerning the thromboprophylaxis methods, a further finding was the underuse of mechanical thromboprophylaxis strategies in medical and surgical patients. We found that 3.3% of patients received mechanical thromboprophylaxis due to elevated bleeding risk, which was the only possible prophylactic measure in such patients. Mechanical methods of thromboprophylaxis include graduated compression stockings (GCS), intermittent pneumatic compression devices, and venous foot pumps [17], and their availability is crucial in thromboprophylaxis strategies.

VTE prophylaxis in medical patients

Regarding medical patients, we found that 40.0% were categorized as high risk for VTE, according to the PPS, similar to other reports in Portuguese hospitals [13,22] and in line with other worldwide studies [7,8,13].

Venous thromboembolism prophylaxis was offered to 77.3% of medical patients, but detailed evaluation shows an overall rate of appropriate thromboprophylaxis in 52.0% of medical patients. On the one hand, a relatively high percentage of high-risk patients received thromboprophylaxis (86.6%), both mechanical and/or pharmacological strategies. This is substantially better than described in previous studies, including Portuguese publications [7,8,13,23], probably emphasizing an increasing awareness of this topic. On the other hand, the overall rate of inappropriate prophylaxis in low-risk medical patients was 73.3%. In 68.9%, inappropriate pharmacological prophylaxis was administered, leading to an increased risk of bleeding events and costs. This finding is consistent with other studies that have shown high use of prophylaxis in low-risk patients [7,13]. These results could reflect either a clinical judgment of real risks and, if so, a clinically correct decision or an incorrect risk assessment [7,13].

All VTE events were symptomatic, one in-hospital VTE event was reported (1.3%) in a medical patient with low VTE risk, which is higher when compared with other studies [8,23]. The low sample size in this study makes it difficult to make conclusions concerning this point. Nevertheless, thromboembolism prophylaxis is a medical practice, and, as with others, clinical judgement ought to influence medical decisions alongside the risk scores. Two medical patients developed VTE events following discharge. Interestingly, both had an in-hospital low risk for VTE. In fact, hospitalized medical patients may have risk factors for VTE that persist for weeks to months after hospital discharge [17,24]. The benefit of extended-duration prophylaxis for certain surgical patients has been established in the literature, and the practice is generally accepted. However, there are very limited studies for medical patients [25]. Some studies that assessed extended thromboprophylaxis in medical patients found no clear clinical benefit; any demonstrated efficacy was outweighed by the significantly increased risk of major bleeding [1]. Further investigation is necessary to clarify the benefits of extended prophylaxis [24].

It is important to note that some thrombotic and bleeding risk factors may coexist, so a combination of risk assessment and clinical judgement should be carefully applied. Regarding bleeding events, 2 medical patients (2.7%) had CRNMB. These patients had received pharmacological thromboprophylaxis despite being considered at low risk for VTE. When compared to other studies, our results are similar or lower [13]. Nonetheless, a bigger sample would be necessary to make this comparison. This emphasizes the importance of the correct application of pharmacological thromboprophylaxis.

VTE prophylaxis in surgical patients

Regarding surgical patients, the results were somewhat unsatisfactory. When compared to the ENDORSE study, we pinpoint a lower rate of patients under adequate prophylaxis (42.0% in our study versus 58.5% in the ENDORSE study), however, our results are far better than those from some international studies, like DissolVE-2, which reports a 10.3% rate of risk-patients receiving adequate prophylaxis [8,13]. In addition, the rate of patients undergoing inadequate prophylaxis was 58.0%, well above expectations, compared to other studies reporting rates inferior to 40.0% [13]. It is important to point out the low adherence to mechanical thromboprophylaxis, indicated in patients with a low VTE risk, but only applied in elevated bleeding risk individuals. 

Concerning surgical patients with high VTE risk, our results showed a lower appropriate thromboprophylaxis strategy (42.0%) because most are only under a pharmacological strategy rather than a combination of pharmacological thromboprophylaxis and GCS. Nevertheless, we believe that this strategy is not inappropriate, as studies are showing that in patients submitted to elective surgical procedures and assessed to be at moderate or high risk of VTE, pharmacological thromboprophylaxis alone seems to be non-inferior to the combination of pharmacological thromboprophylaxis and GCS [26].

Still, we shall consider the percentage of patients under in-hospital pharmacological thromboprophylaxis (83.0% of surgical patients) encouraging, as it reflects an increased awareness of surgeons with VTE prevention. Two surgical patients were reported to have VTE who were not under thromboprophylaxis despite having indications. Awareness about VTE prevention among surgeons must not be forgotten to provide better care to our patients.

About major bleeding events, the three reported events in surgical patients came from the group in which pharmacological thromboprophylaxis was not initiated due to high hemorrhagic risk, despite indicating to. These data enhance the difficulty of managing these high-risk patients and suggest thromboprophylaxis safety. 

Strengths and limitations

This is one of the most recent observational studies regarding VTE prophylaxis in Portugal of the past ten years. Also, it includes a diverse population since it occurred during a pandemic and presents this reality. Another strength of this study is that it included evaluation of events 3 months after discharge.

We have identified some limitations of our study that may influence our results. Firstly, the fact that it was conducted in a single center over a short period with a small number of patients. This fact impacted the number of events and their relevance in the global sample. Moreover, our data was collected based on patient information and status at admission, therefore, changes that occurred within the period of hospitalization were not taken into consideration. Finally, our study occurred during the COVID-19 pandemic period, which influenced not only the overall risk of VTE but also thromboprophylactic measures. Besides, there were less elective surgical admissions and, on the other hand, less urgent admissions in medical patients.

Concerning the two cases that occurred in SARS-CoV-2-positive patients, a possible relation between the events may be inferred since it has been well-established as a risk factor [27]. The true impact of this pandemic and the inclusion of SARS-CoV-2-positive patients in this population must be clarified in further larger studies.

## Conclusions

Hospitalization for acute illness is associated with an increased risk of VTE, being the leading preventable cause of in-hospital death. Venous thromboembolism risk assessment is essential for the accurate identification of patients who might benefit from VTE prophylaxis and consequent prevention strategies implementation.

This study provided a real perspective on clinical practice of VTE risk assessment and prophylaxis in our hospital. It showed that although a considerable proportion of medical and surgical patients are at risk of VTE, appropriated VTE prophylaxis is low, which was both in the context of underuse or excessive prophylactic measures. These findings highlight the need for better thromboprophylaxis protocol implementation.
